# Wounds of the Present War

**Published:** 1915-05

**Authors:** 


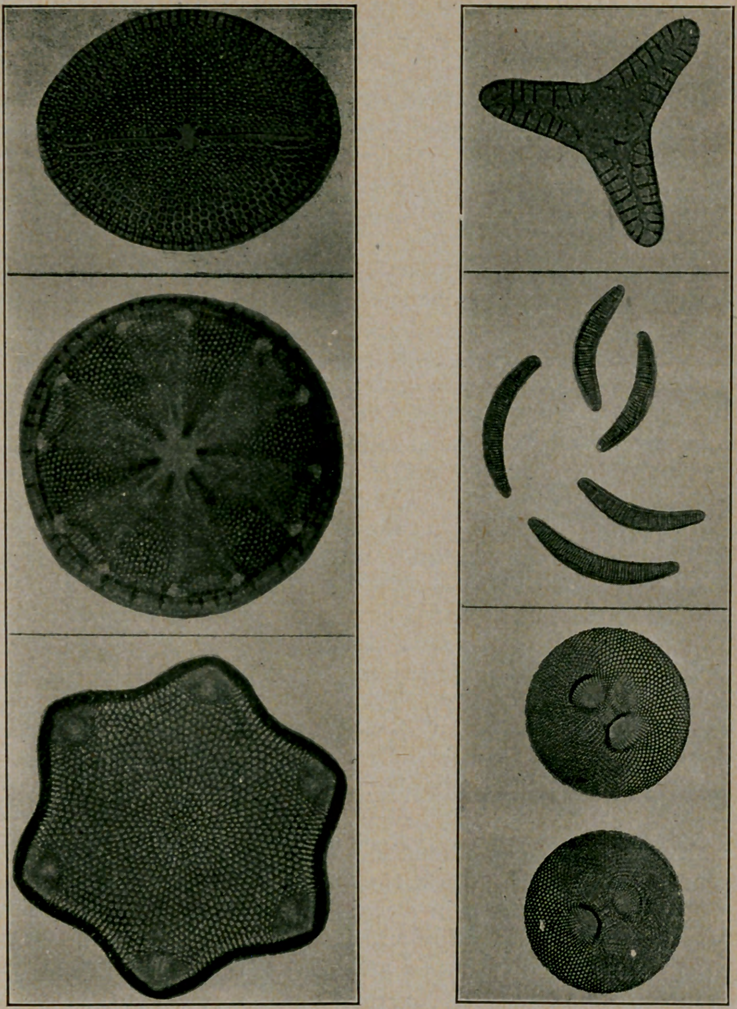

**Wounds of the Present War.** G. H. Makins, Lancet. As compared with the Boer war and earlier campaigns, there is a disproportionate number of machine gun bullet wounds in the class of bullet wounds, and a disproportionate number of shrapnel and shell wounds as compared with all wounds from small arms. The serious wounds, however, resemble closely, the water-color drawings of Peninsular and Crimean wounds, made by Sir Charles Bell, at that period. Wounds by small calibre bullets usually remain uninfected while those by shrapnel and shell usually suppurate, though with applications of iodine 1:128, suppurating wounds usually clear up with surprising rapidity and lack of serious complications, if affecting non-vital soft parts. Except that the openings are slightly larger, the rifle bullet wounds do not differ essentially from those made by Lee-Metford or dome-shaped Mauser bullets. Nerve and vascular injuries are rare. Differences of effect according to velocity are mentioned, as has already been well described. Wounds of exit of slow projectiles, especially when bone has been struck, are large, often with large protruding masses of muscle, comminuted bone, etc. Lodgement both of bullets and shrapnel balls is common and contour wounds of the chest and head are not rare. A case of five separate wounds from machine gun bullets and one of 34 wounds from shrapnel balls, are cited. Multiple wounds, even if no one is especially serious produce great shock. On the whole, most cases recover promptly and even the bodily and mental conditions to be expected from exposure in the trenches, are not serious. If men have lain for some time after receiving a wound, gaseous gangrene and tetanus are likely to occur, from soil contamination, whereas the dusty, mainly unoccupied soil of the Boer campaign, did not contain the corresponding germs. In probable cases, tetanus antitoxin is used as a routine prophylactic. Tetanus runs a rapid course but usually without severe spasms. Wounds of the face, jaw, palate, etc., are common, on account of the relatively greater protection of other parts of the body in the trenches. Transportation difficulties were great at the beginning of the campaign, owing to the rapid movement of large bodies of men and exposed fighting. At present, the problem is simpler.

